# Response of skin temperature, blood ammonia and lactate during incremental exercise until exhaustion in elite athletes

**DOI:** 10.1038/s41598-024-52374-z

**Published:** 2024-01-26

**Authors:** Paweł Korman, Krzysztof Kusy, Anna Straburzyńska-Lupa, Adam Kantanista, Manuel Sillero Quintana, Jacek Zieliński

**Affiliations:** 1 Department of Physical Therapy and Sports Recovery, Faculty of Health Sciences, Poznan University of Physical Education, 61-871 Poznań, Poland; 2 Department of Athletics, Strength and Conditioning, Faculty of Sport Sciences, Poznan University of Physical Education, 61-871 Poznań, Poland; 3 Department of Physical Education and Lifelong Sports, Faculty of Sport Sciences, Poznan University of Physical Education, 61-871 Poznań, Poland; 4 Faculty of Physical Activity and Sports Sciences (INEF), Universidad Politécnica de Madrid, 28040 Madrid, Spain

**Keywords:** Imaging and sensing, Vasodilation, Biomarkers

## Abstract

The study aimed to evaluate the lower limb skin temperature (Tsk) and blood concentrations of lactate (LA) and ammonia (NH_3_) during exercise and recovery. Eleven elite sprint athletes (25 ± 3.4 yrs) and 11 elite endurance athletes (24.45 ± 5.4 yrs) performed an incremental running test until exhaustion. Body composition was estimated using the DXA method. Thermograms of the anterior and posterior surfaces of the lower limbs were recorded at rest, before each test stage (every 3 min, starting from 10 km h^−1^ and increasing by 2 km h^−1^), and in the 5th, 10th, 15th, 20th, and 30th minute of recovery. Endurance athletes had a higher maximum oxygen uptake than sprint athletes (5.0 ± 0.7 vs 4.3 ± 0.4 l·kg^−1^, *p* = 0.018), lower percentage of lean content (79 ± 2 vs 83 ± 2%, *p* < 0.001), and a higher percentage of fat content in the lower limbs (17 ± 2 vs 12 ± 2%, *p* < 0.001). In both groups, a significant decrease in Tsk was observed compared to resting value (endurance athletes—31.5 ± 0.6 °C; sprint athletes—32.3 ± 0.6 °C), during exercise (*p* < 0.001) and rewarming during recovery (*p* < 0.001). However, endurance athletes had a lower Tsk than sprint athletes at the exhaustion point (30.0 ± 1.1 vs 31.6 ± 0.8 °C, *p* < 0.05) and the pattern of change in Tsk differed between groups (*p* < 0.001). Tsk in the endurance athletes group decreased throughout the exercise protocol and returned more rapidly to initial values during recovery, while Tsk in the sprint group stabilised between moderate intensity and exhaustion, recovering more slowly after exercise. Both LA (endurance athletes—max 10.2 ± 1.5; sprint athletes—max 10.1 ± 1.4 mmol⋅L^−1^, *p* < 0.001) and NH_3_ (endurance athletes—max 75.6 ± 11.5; sprint athletes—max 76.7 ± 9.0 mmol⋅L^−1^, *p* < 0.001) increased during exercise and decreased during recovery (*p* < 0.001). During exercise, lower levels and slower increases in LA were observed during exercise in the endurance athletes’ group (*p* < 0.05). A negative correlation was revealed between Tsk and fat percentage (r = −0.43 to −0.71, *p* < 0.05). Tsk was positively correlated with LA during recovery (r = 0.43 to 0.48, *p* < 0.05), and negatively during recovery (r = −0.45 to −0.54, *p* < 0.05). Differences between groups in maximum aerobic capacity, the pattern of change in Tsk, and the correlation between Tsk and LA suggest that individuals who decrease less Tsk during exercise and higher Tsk during recovery are those with better aerobic capacity. In addition, athletes with less body fat dissipate heat from their tissues more efficiently.

## Introduction

Knowing the optimal physiological parameters related to exercise intensity and recovery efficiency in highly trained athletes is of great interest to scientists and sports coaches. Lactate (LA) and ammonia (NH_3_) are commonly used biomarkers that have been extensively studied during various types of exercise^1–4^. Lactate is formed when adenosine triphosphate (ATP) is generated under oxygen deficiency conditions^5^. Increased LA concentration is an indicator of anaerobic muscle metabolism and impaired muscle function during exercise^5^. NH_3_ in the blood is a direct product of adenosine monophosphate (AMP) degradation^6^ and can be used as an extracellular marker of ATP stores in skeletal muscle^7^. Therefore, the increases and the maximum concentrations of LA and NH_3_ in the blood are considered good indicators to monitor the training process^4,8,9^. The levels of LA and NH_3_ during exercise and the speed of their use indicate the training status of untrained individuals, and in the case of trained athletes, they can indicate the type of exercise performed^8–10^.

One of the mechanisms strongly activated during exercise is thermoregulation manifested by changes in skin temperature (Tsk). Depending on the type of physical effort, changes in Tsk may have different patterns^11^. For constant intensity endurance exercise, there is an initial decrease in Tsk followed by a plateau phase^12^ or an immediate return to baseline or higher Tsk levels until the exercise is complete^11^. In the case of resistance exercise, researchers observed an increase in Tsk in exercise areas^11^. Most research groups observed that during exercise of increasing intensity, Tsk gradually decreases and returns to baseline values during the initial post-exercise recovery period^13–15^. In other cases, such as strength exercise, Tsk decreases or remains constant after exercise to increase during long-term recovery^16,17^. It can be assumed that the Tsk response varies depending on the type of effort, but at the same time.

Body composition is among the many factors that affect Tsk at rest and during exercise. So far, a relationship between body mass index (BMI) and Tsk has been observed, that is, a lower BMI is associated with a higher Tsk and a higher BMI with a lower Tsk^18^. In particular, the effects of body fat and fat-free mass have been studied, and it is now known that the lower the content of adipose tissue, the higher the Tsk^19–21^. Only a few studies have been devoted to the relationship between fat tissue and Tsk during exercise^22,23^. It was shown that Tsk during exercise depends on body components, the proportions of which vary depending on the sports discipline. To date, there has been a lack of studies to assess the relationship between body composition and Tsk during increased intensity exercise and post-exercise recovery in athletes of different sports.

Because exercise is accompanied by changes in blood lactate^24^, ammonia levels^8^, and Tsk^16^, these parameters could be related to each other. Previously, several studies have been published on the correlation of Tsk with LA^3,25,26^, but not with NH_3_. Apart from the obvious co-occurrence of decreasing Tsk and increasing LA blood levels during exercise and the opposite trend during recovery, there are no data on the relationship between these indicators during the increasing intensity of exercise.

The aim of this study was to determine the change in Tsk and blood concentrations of two exercise biomarkers (LA, NH3) and the correlations between them during graded exercise and recovery in athletes of different sports. To our knowledge, this is the first study to monitor variations in Tsk and exercise biomarkers (LA, NH3) levels in blood at baseline, during graded exercise, and after exercise with multiple samples in elite athletes who have different training backgrounds and metabolic responses. We hypothesise that (i) there are differences between sports in Tsk patterns and in the blood concentration of exercise biomarkers, (ii) there is a correlation between Tsk and exercise biomarkers, and (iii) the level of Tsk depends on body composition.

## Material and methods

### Experimental approach to the problem

Elite athletes representing two distinct sports disciplines and related physiological adaptations (speed-power vs endurance) participated in the study. The main procedures included repeated blood sampling and surface temperature measurements at rest, during the progressive exercise test, and during the 30-min recovery period.

### Participants

The study included 22 highly trained male athletes, all members of the Polish national teams. They were sprint athletes (n = 11, age range 21 to 31 years), practising competitive sport for 9.5 ± 2.6 years, and endurance athletes (n = 11, age range 15 to 34 years) represented by triathletes and long-distance runners, practising sport for 10.2 ± 2.9 years. Detailed characteristics are shown in Table 1. The inclusion criteria was training professionally in a given discipline for at least the last 5 years and a current call-up to the national team. Exclusion criteria included any lower extremity injury in the last month, the use of creams and ointments on the skin of the lower limbs, any vigorous physical exertion on the day of the examination, the undergoing physical therapy, and any signs of health indisposition, especially affecting body temperature above 37.5 °C (measured before tests with a Geratherm non-Contact—Germany pyrometer). The study was carried out according to relevant guidelines and regulations of the Declaration of Helsinki and was approved by the Ethical Committee of the Poznan University of Medical Sciences. All participants received a detailed explanation about the objective, testing procedures, risks and benefits of the research, and gave their written consent before the examination.

**Table 1:** Basic characteristics of athletic groups.

	Endurance athletes (n = 11)	Sprint athletes (n = 11)	*p*	η^2^
Age (yr)	24.5 ± 5.4	25 ± 3.4	0.781	0.004
Training Experience (yr)	10.2 ± 2.9	9.6 ± 2.6	0.592	0.015
Height (cm)	181.6 ± 5.9	184.8 ± 4,9	0.182	0.087
Weight (kg)	74.4 ± 7.9	78.7 ± 6.1	0.172	0.091
BMI (kg·m^-2^)	22.5 ± 1.9	23 ± 0.9	0.491	0.024
SMM (kg)	32.7 ± 3.8	37.6 ± 3.6	0.005*	0.334
Total Fat Mass (kg)	11.8 ± 2.4	9.6 ± 1.5	0.020*	0.240
Legs Lean Mass (kg)	20.7 ± 2.5	23.7 ± 2.4	0.009*	0.291
Legs Lean Mass (%)	79 ± 2	83 ± 2	< 0.001*	0.603
Legs Fat Mass (kg)	4.4 ± 0.9	3.4 ± 0.5	0.003*	0.361
Legs Fat Mass (%)	17 ± 2	12 ± 2	< 0.001*	0.604
HR_max_ (bpm)	191.5 ± 8.4	190.7 ± 8.7	0.844	0.002
V̇O_2_max (l·kg^−1^)	5.0 ± 0.7	4.3 ± 0.4	0.018*	0.249
V̇O_2_max (ml·kg^−1^·min^−1^)	66.5 ± 4.4	55.0 ± 4.4	< 0.001*	0.650
V̇O_2_max (ml·kg SMM^−1^·min^−1^)	151.7 ± 11.3	115.2 ± 9.0	< 0.001*	0.779

*Significantly different between the groups.

Abbreviations: BMI, body mass index; HR, heart rate; SMM, skeletal muscle mass; V̇O_2_max, maximal oxygen uptake.

### Procedure

All tests were carried out at the Human Movement Laboratory of Poznan University of Physical Education. The laboratory room was air-conditioned and temperature and humidity (21.0 ± 1.0 °C and 60 ± 5%, respectively) were monitored using a thermohygrometer (Onset HOBO Temp/RH 2.5% Data Logger, UX100-011—USA). Athletes arrived at a laboratory in the postabsorptive state, fasting for at least 12 h. The first measurement was a whole-body scan to assess body composition. Subsequently, the athletes could have a light meal consisting of a sandwich and up to 0.5 L of water, without coffee, tea, energy drinks, or ergogenic supplements. Then, the participants performed an incremental treadmill test until exhaustion accompanied by blood sampling and measurement of Tsk of the lower limbs.

### Anthropometric and physiological variables

On the day of the experiment, weight and height were measured using a digital stadiometer (SECA 285, SECA, Hamburg, Germany). Dual X-ray absorptiometry method, using the Lunar Prodigy Pro DXA device (GE Healthcare, Madison, WI, USA) and enCORE v. 16 SP1 software was used for body composition analysis. The device was calibrated using a phantom according to the manufacturer's guidelines. The athletes were tested in their underwear, without any metal objects or jewellery. All DXA scans were performed and analysed by the same trained technician according to the manufacturer's protocols. Skeletal muscle mass (SMM) was calculated on regression models according to Kim et al.^27^.

### Exercise test and respiratory parameters

A progressive exercise treadmill test (H/P Cosmos Pulsar, Sports & Medical, Nussdorf-Traunstein, Germany) was used to track a wide range of intensity of exercise^28^. After 3 min of standing on the treadmill, the participants walked at a speed of 4 km h^−1^ for the first 3 min, then it was increased to 8 km/h^−1^. After that point, the treadmill speed increased by 2 km h^−1^ every 3 min until volitional exhaustion. Blood samples were taken at the end of each 3-min stage, starting from a speed of 10 km h^−1^. Respiratory parameters were measured with an ergospirometer (Cortex Metamax 3B R2, Leipzig, Germany) and analysed using Metasoft Studio v. 5.1.0 Software (Cortex-Metamax 3B R2; Cortex Biophysik, Leipzig, Germany). The Polar Bluetooth Smart HR monitor H6 (Polar Electro Oy, Kempele, Finland) was used to monitor heart rate (HR). Maximal oxygen uptake (VO_2_max) was considered achieved if at least three of the following criteria were met: (i) a plateau in V̇O_2_ despite an increase in speed and minute ventilation; (ii) blood lactate concentration ≥ 9 mmol⋅L^−1^; (iii) respiratory exchange ratio ≥ 1.10; and (iv) heart rate above 95% maximum heart rate predicted by age^29^. All participants were familiar with the exercise protocol as they had previously done.

### Thermographic measurement

**Figure 1:**
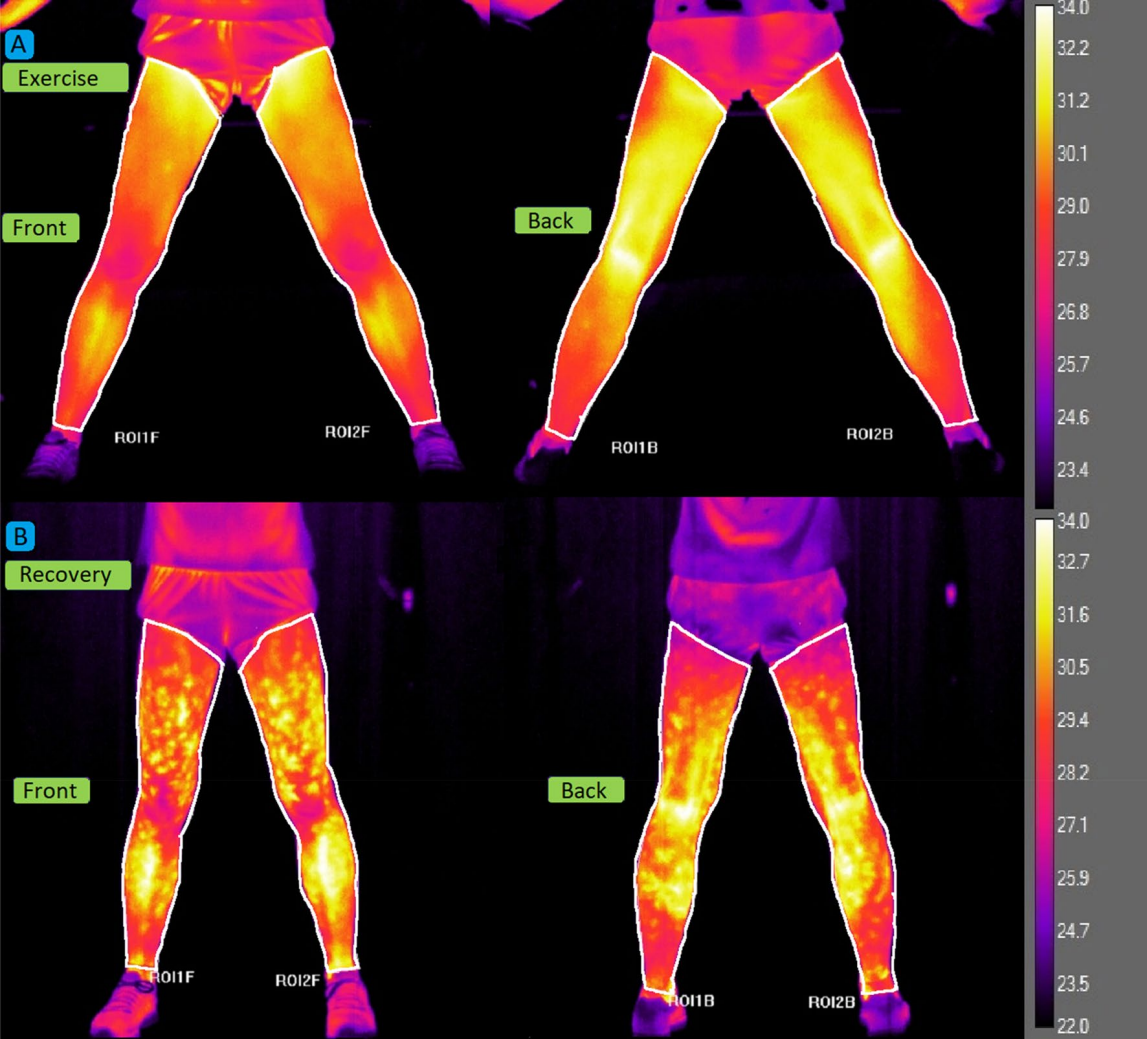
Examples of Regions of Interest (ROI) selection: (**A**) Exercise: before and during exercise (on the treadmill), (**B**) Recovery: during recovery (out of the treadmill).

The athletes were wearing only light sportswear that exposed their lower limbs. It was forbidden to use ointments, creams, or lotions 24 h before the test. The uncooled FLIR SC640 IR camera (FLIR Systems Inc., SC640 model, Sweden) with noise-equivalent temperature difference (NETD) < 30 mK, 640 × 480 pixels of resolution and a temperature accuracy of ± 2% was used to record the thermograms. The camera was positioned on a tripod 1 m above the ground and 2 m from the participant. The TISEM checklist has been used to ensure the reliability of thermal imaging research and analysis^30^. Approximately 20 min before the start of the test, the participants were acclimatised in the laboratory room in a sitting position so that the lower limbs did not touch each other and had minimal contact with the seat. Infrared images of the anterior and posterior surfaces of the lower limbs were taken at rest, at the end of each 3-min stage above the speed of 10 km·h^−1^, immediately after exercise and 5, 10, 15, 20, and 30 min into the recovery period. For the temperature analysis, regions of interest (ROI) were established (Fig. 1). The mean and standard deviation of the surface Tsk of the front and back sides of the lower limbs were calculated using dedicated software (Thermacam Researcher Pro 2.10 software, FLIR, Wilsonville, Oregon, USA). The front ROI covered the area between the inguinal ligament and the talocrural region. The ROI on the back of the lower limbs covered the area between the gluteus sulcus and the talocrural region.

### Blood sampling

A catheter (BD Venflon Pro 1.3 3 32 mm; Becton Dickinson, Helsingborg, Sweden) was inserted into the antecubital vein with isotonic saline (0.9% NaCl). Blood samples were collected at the same time as the infrared images were taken. Later, a 2.7 ml blood sample was taken in 2 monovettes (S-Monovette 2.7 ml KE; Sarstedt, Nümbrecht, Germany), one with a lithium anticoagulant (heparin) and another containing an anticoagulant (EDTA). After each sampling, the catheter was flushed with isotonic saline (0.9% NaCl) after each sampling to maintain its patency.

### Lactate and ammonia

The Biosen C-line device (EKF diagnostic GmbH, Barleben, Germany) was used to measure the LA concentration. For this procedure, 20 ml of whole blood was placed in a capillary. Measurement precision (CV) was 1.5% for a concentration of 12 mmol·l^−1^. The PocketChem BA PA-4140 device (Arkay, Kyoto, Japan) with a measuring range of 8‒285 mmol l^−1^ and CV of 2.3.% was used to determine the level of NH3. Using a pipette, 20 ml of blood was applied to the test strip (Ammonia Test Kit II; Arkay), which was then placed in the device.

### Statistical analysis

A one-way analysis of variance (ANOVA) was used to calculate the differences in descriptive variables. Two-way repeated measures ANOVA was performed to test the main effects of the test stage and group and their interactions for the response of Tsk, LA, and NH3 to exercise. The sample size was estimated based on the assumption that the effect size will be at least medium. Using an α-level of 0.05, a power (1-β) of 0.8, and *η*^2^ = 0.14 (large), it was calculated that in total at least 16 participants are required to detect a significant change or differences in temperature, LA, and NH_3_ concentration (G*Power; Heinrich-Heine-Universitat Dusseldorf, Dusseldorf, Germany). The Mauchly’s sphericity test was used and the Greenhouse–Geisser correction was applied if the assumption of sphericity was violated. The effect size for the ANOVA was interpreted as small (0.01), medium (0.06), or large (0.14). If a significant main effect or interaction was found, post hoc Bonferroni tests were performed. Pearson’s correlation coefficients (r) were used to describe the relationship between biomarker concentrations and Tsk in the combined group of athletes at each stage of exercise and in recovery, and were defined as small (0.1), medium (0.3), or large (0.5). The significance level for all statistical analyses was established at *p* < 0.05. All values were presented as mean ± standard deviation. The calculations were made using the STATISTICA 12.0 software package (Stat-Soft, Tulsa, OK, USA).

### Ethical approval

Research was approved by Ethical Committee at the Poznan University of Medical Sciences.

### Consent to participate

Written informed consent was obtained from all participants.

## Results

### Subjects description

The study groups differed from each other in several parameters. Sprint athletes had a significantly higher SMM and less leg fat mass compared to the endurance athletes group. Differences also included higher V̇O_2_max in endurance athletes, expressed as both absolute (l·kg^−1^) and relative (ml·kg^−1^·min^−1^ and ml·kg SMM^−1^·min^−1^) values. There were no significant differences between the groups with respect to the remaining parameters. All detailed baseline anthropometric and physical characteristics are presented in Table 1.

**Figure 2:**
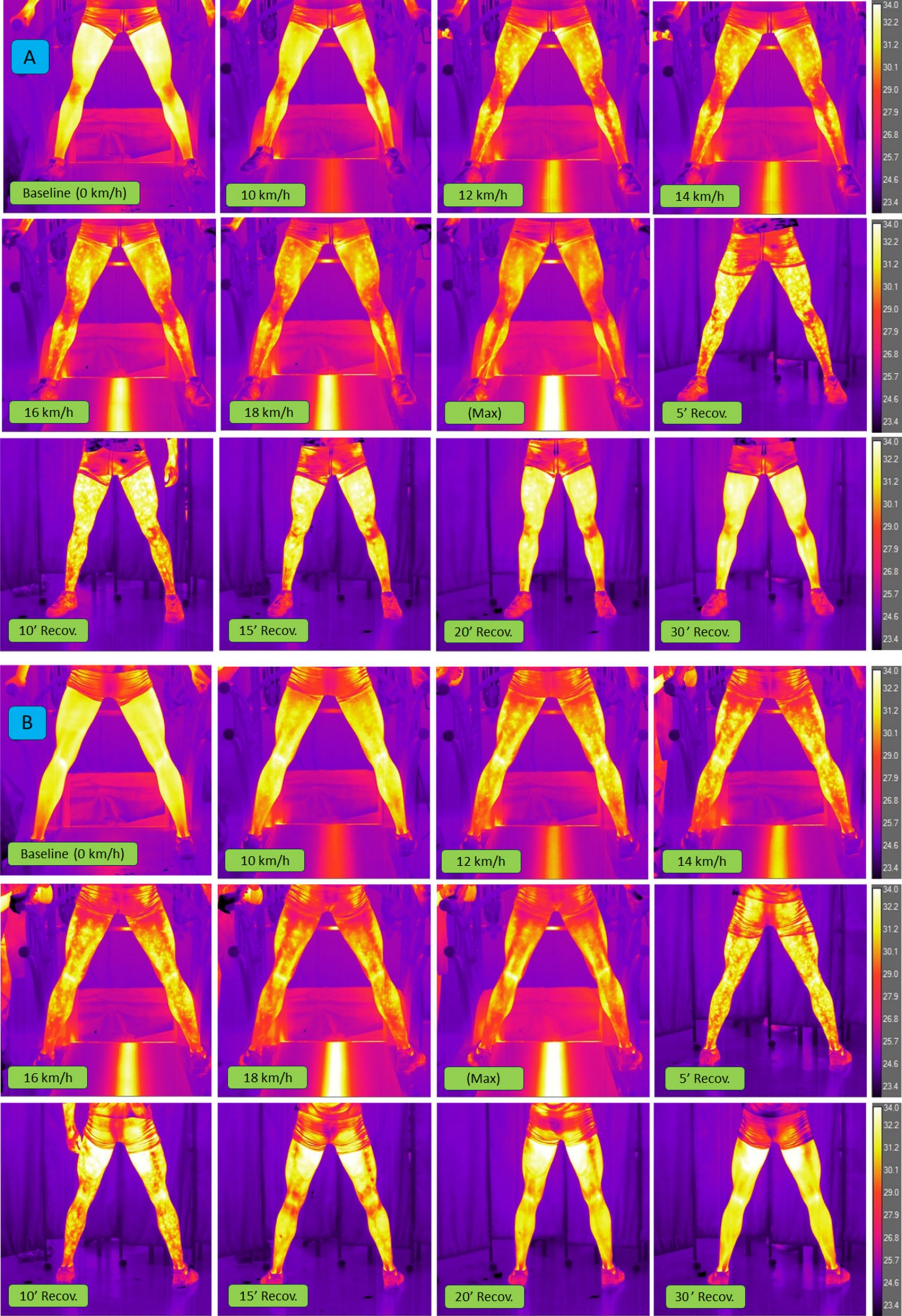
Example of the thermal response of a member of the endurance athletes group during the whole data protocol in the anterior view (**A**) and the in posterior view (**B**).

Figures 2A,B show a full series of thermograms of an athlete from the endurance athletes’ group to better visualise the Tsk response throughout the test protocol.

**Figure 3:**
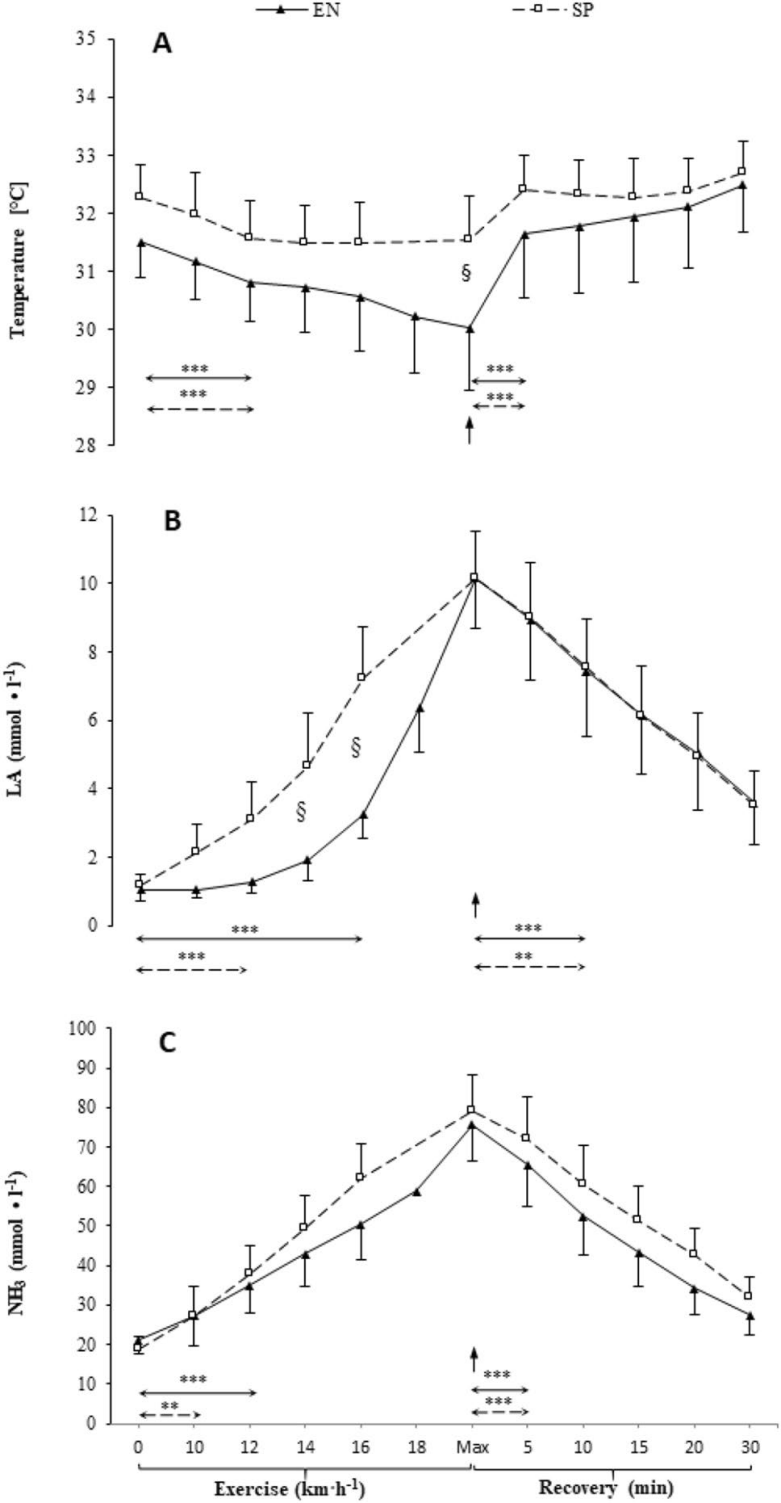
Skin temperature (Tsk, panel A), venous plasma lactate (LA, panel B) and, ammonia (NH_3_, panel C) concentrations at rest, during incremental treadmill exercise test until exhaustion (Max), and during recovery in endurance athletes (EN) and sprint athletes (SP). Data are mean values ± SD. Vertical arrows indicate the point of volitional exhaustion. Horizontal arrows indicate the first significant difference from baseline or exhaustion within groups. ***p < 0.001, **p < 0.01 ‒ significant differences between sampling points; § *p* < 0.05 ‒ significant differences between groups at the same sampling point.

The course of changes in mean levels of Tsk, LA, and NH_3_ levels during the incremental exercise test and 30-min recovery is shown in Fig. 1. In the case of Tsk (Fig. 3A), there was a significant main effect for the test stages (*p* < 0.001, η^2^ = 0.69). The resting temperature was 31.5 ± 0.6 °C for endurance athletes and 32.3 ± 0.6 °C for sprint athletes. In all participants, a decrease in Tsk was observed at the beginning of the exercise. The first significant decrease in Tsk occurred at the speed of 12 km h^−1^ (*p* < 0.001). When exhausted, the endurance athletes reached 30.0 ± 1.1 °C and the sprint athletes reached 31.6 ± 0.8 °C. Once the exercise was over (exhaustion), the Tsk began to rise, most rapidly in the first 5 min of recovery, until the end of the observation in the 30th minute after exercise. A statistically significant group effect was also noted (*p* < 0.05, η^2^ = 0.22), i.e. the sprint group was characterised by higher Tsk levels than endurance athletes throughout the test, with a significant difference at the end of the exercise (*p* < 0.05). There was a statistically significant effect of the interaction group*test stage (*p* < 0.001, η2 = 0.13). Although Tsk in the endurance group decreased throughout the whole exercise protocol, Tsk in the sprint group stabilized between 12 km h^−1^ and exhaustion. All endurance athletes completed the test at the 20 km h^−1^ stage, ten sprint athletes completed the 18 km h^−1^ stage, and one the 20 km h^−1^ stage.

In the case of LA (Fig. 3B), there was a significant main effect for the test stages (*p* < 0.001, η^2^ = 0.91). In both groups, the LA levels continuously increased from the beginning of the exercise. The first significant increase in LA occurred at the speed of 12 km h^−1^ in the sprint group and at 16 km h^−1^ (*p* < 0.001) in the endurance group. LA increased until the end of the test. From then on, it decreased significantly until the end of the observation in both groups. A statistically significant group effect was noted (*p* < 0.05, η^2^ = 0.23), that is, sprinters were characterised by a higher LA level than endurance athletes in the entire range of intensity of exercise, especially at the speed of 16 and 18 km h^−1^ (*p* < 0.05). There was also a statistically significant interaction between groups*test stages interaction (*p* < 0.001, η^2^ = 0.35). In the sprint group, the increase in LA during exercise was almost linear, while endurance athletes exhibited a curvilinear relationship between lactate and exercise intensity with a slower increase at low intensity.

In the case of NH_3_ (Fig. 3C), there was a significant main effect for the test stages (*p* < 0.001, η^2^ = 0.94). In both groups, the NH_3_ level continuously increased from the beginning of exercise. The first significant increase in NH_3_ occurred at the speed of 10 km h^−1^ in the sprint group and at 12 km h^−1^ in the endurance group. The NH3 concentration increased until the end of the test. From then on, the NH_3_ level decreased until the end of the observation in both groups. There was no group effect (*p* = 0.279, η^2^ = 0.06). There was a statistically significant group*test stage interaction (*p* < 0.05, η^2^ = 0.13): the increase in NH_3_ concentration in the last phase of the test was slower in the endurance group.

There were positive moderate correlations between Tsk and LA for the speed range of 10‒16 km h^−1^ (r = 0.43‒0.48, *p* < 0.05) and negative Tsk-LA correlations between the 10th and 30th minute of recovery (r ranging from −0.54 to −0.45, *p* < 0.05). There were no significant correlations between Tsk and NH_3_ and between LA and NH_3_ at all stages of the test. Significant positive correlations (r = 0.46–0.74, *p* < 0.05) occurred between Tsk and the percentage content of lean mass in all test stages. A significant negative correlation (r ranging from −0.71 to −0.43, *p* < 0.05) also occurred between Tsk and the percentage of fat content at all stages of the test. The correlation between Tsk and SMM (r = 0.44, *p* < 0.05) occurred only in the first measurement, before exercise.

## Discussion

To our knowledge, this is the first study to track changes in Tsk and exercise biomarkers (LA, NH_3_) concentration in blood at rest, during incremental exercise, and during the recovery period with many sampling points in highly trained athletes representing opposite training profiles and resulting metabolic adaptations. Our main findings were that the pattern of change in Tsk and LA concentration levels depends on the sports-related training profile. The endurance athletes had a lower Tsk observed at the end of the exercise and a slower increase in LA levels during exercise than the sprint athletes. It was also observed that, regardless of the training profile, a higher Tsk corresponded to a higher LA concentration during exercise. On the contrary, during recovery, a higher Tsk corresponded to a lower LA concentration. No significant relationship of Tsk with NH_3_ was observed. Furthermore, Tsk was positively correlated with lean mass and inversely correlated with fat content.

In our study, we observed a significant decrease in Tsk during exercise and rapid rewarming during recovery. This pattern was also observed in other studies. Tanda^14^ indicated that long-distance runners showed a decrease in Tsk in the initial phase of running effort, regardless of the type of work performed (in the field or on a treadmill) and environmental conditions (outdoor or indoor). The Tsk drop was more pronounced until the end of the test with graded load (from 6 to 13.5 km h^−1^) than with constant load (12 km h^−1^). In the study by Oliveira et al.^31^, a decrease in Tsk was also observed in distant areas of the body. Participants performed an incremental test on the upper limb ergometer; however, Tsk decreased during exercise in the lower limbs and trunk to return to initial values during recovery. This was confirmed by Hillen et al.^11^ in a review in which they noted that all studies on endurance and incremental exercise reported a measurable decrease in Tsk. During constant-intensity endurance exercise, Tsk drops initially and stabilises after a few minutes. Additionally, Fernandes Ade et al.^32^ observed a decrease in Tsk of distant areas during aerobic exercise at an intensity of 60% V̇O_2_max, followed by rewarming during recovery. The gradual increase in exercise intensity causes a further decrease in Tsk. The difference in Tsk change between constant-intensity and incremental exercise reveals the dependence of Tsk on exercise intensity. Other studies based on high-intensity or incremental exercise, analysing cycling^15^, strength training^17^, or rowing^33^, confirm a similar course of Tsk.

The plausible mechanism for the decrease in Tsk during incremental exercise involves sympathetic noradrenergic nerve activity and its effects on cutaneous arterial vasoconstriction^34^. The sympathetic noradrenergic vasoconstrictor nerve, controlled by the rostral medulla oblongata and medulla oblongata preoptic area, initiates the release of norepinephrine and neuropeptide Y, which activate the α-adrenergic receptors of smooth muscles of the vascular skin^35^. This leads to contraction of the cutaneous blood vessels and to the consequent redistribution of blood volume to activated organs^36^, for example skeletal muscles^37^. If the temperature of the deep tissue in the exercising or recovering muscle exceeds a certain level, cholinergic nerve transmission activates the nonadrenergic vasodilator system^38^. It leads to active vasodilation of skin vessels for thermoregulation^34,38^. It seems that changes in Tsk during exercise and at rest are more pronounced and faster in trained people than in untrained individuals and may indicate the training status^39^. In our study, there was a difference in the evolution of Tsk between the groups with significantly lower temperature in endurance athletes at the end of the exercise. Study participants were highly trained and, therefore, similar in terms of training status but different in terms of specific physiological characteristics and adaptations. We suppose that the differences in Tsk levels and the course obtained in our study are due to these adaptations, including the amount of SMM or body fat, which is explained later.

Of course, a better adaptation to exercise in the endurance athletes group is a more likely cause, for example, in terms of sweating. Especially, from 12 km h^−1^ we did not observe a clear drop in Tsk in the sprint group compared to the endurance athletes group. Smith and Havenith^40^ indicated that regional sweat rates do not correlate with Tsk. Therefore, it cannot be ruled out that measurements of the whole-body surface temperature would give a different picture of the relationships. It is also worth paying attention to the role of perforator vessels whose operation in the thermoregulation process is visible in thermography as hot spots. Brito et al.^41^ observed that if the hot spots covered more than 70% of the body area after physical exercise, this was associated with a lower Tsk. The analysis of hot spots was not the aim of this manuscript, but we think that it is worth considering it in future studies to better understand thermoregulatory processes.

The relationship between body composition (especially body fat) and Tsk has been described in previous studies that are consistent with our results. Chudecka et al.^19^ used the bioimpedance technique to assess the effect of adipose tissue on Tsk in adult women. They compared 20 obese women (37.8 ± 2.3% of body fat) with 20 non-obese women (25.7 ± 2.4% of body fat). Women with obesity had lower Tsk values (*p* < 0.05) of Tsk in the arms, thighs, calves, abdomen and lower ribs. Similarly, Reis et al.^42^ studied 50 obese (31.5 ± 4.3% of body fat in the lower limbs) and 50 non-obese men (12.73 ± 3.4% of body fat in the lower limbs) using the DXA method and showed a significantly higher Tsk (*p* < 0.001) in non-obese men. In our study, we did not find any difference in resting Tsk between the groups. This can be explained by the high training status of the participants, which results, among other things, in a unique body composition characterised by high lean mass and low fat tissue compared to the general population in both groups. However, when analysing the combined group of our athletes, we showed a positive correlation between Tsk and lean mass (%) and a negative correlation with leg fat mass (%). It is consistent with other studies. In the study by Neves et al. ^20^ on Tsk in 47 men and 47 women, they observed that the percentage of body fat was negatively correlated with the Tsk of the anterior (r = 0.57, *p* < 0.05) and posterior surface of the lower limbs (r = 0.63, *p* < 0.05) in men and anterior (r = 0.36, *p* < 0.05) and posterior surface of the lower limbs (r = 0.40, *p* < 0.05) in women, respectively. Similarly, Salamunes et al.^21^ observed a negative correlation between the amount of fat tissue and Tsk in the anterior and posterior areas of the lower limbs (r = 0.38 and r = 0.49, *p* < 0.001, respectively), in 123 women. In the case of SMM, apart from the initial measurement, we did not show any correlation with Tsk. This may indicate that, in highly trained athletes, SMM does not seem to be a useful parameter in the context of exercise thermoregulation. Lean mass and fat mass provide better diagnostic possibilities. Although the relationship between BMI and Tsk has already been observed^18,19^, so far we have not found studies that would indicate permanent correlations between Tsk and body composition during exercise and post-exercise recovery.

As expected from previous studies^1–3^, the decrease in Tsk during exercise was accompanied by an increase in LA and vice versa during recovery. Studies showing the relationship between NH_3_ and Tsk during exercise are not available. In the context of changes in Tsk and LA levels during exercise, only a few studies can be indicated. Akimov and Son’Kin^2^ tested 20 athletes performing incremental exercise on the bicycle ergometer and showed a decrease in Tsk with a concomitant increase in LA. However, Tsk was measured on the forehead. Similarly, Adamczyk et al.^3^ studied the relationship between lower limbs temperature and LA in sixteen untrained adults. They monitored parameters only at rest and during a 30-min recovery (9 measurements) after a one-minute bout of vertical jumps. Those authors observed that the mean Tsk of the lower limbs and the blood LA concentration were weakly negatively correlated (r = −0.29, *p* < 0.05). Unfortunately, this correlation was achieved only by combining all 160 measurements for analysis. Similarly to our results, Temfemo et al. ^25^ showed a significant positive correlation (r = 0.69, *p* < 0.001) between Tsk and LA during exercise tests. They tested 18 regional-level soccer players who performed repeated 6-s sprints with increasing load on a cycle ergometer, interspersed with 5-min recovery periods. Their results showed an increase in Tsk with subsequent stages of the test. However, blood sampling for LA took place only 4 min after each sprint, and the highest Tsk value obtained during the 5-min rest between sprints was used. Therefore, it is difficult to determine whether the change in Tsk was a direct effect of exercise or rather post-exercise recovery. Previous studies indicate a decrease, not an increase, in Tsk during incremental exercise^11^.

In our study, due to parallel measurements of Tsk and LA, we observed that during incremental exercise, a lower Tsk was associated with lower LA levels. Previous studies^39,43^ indicated that trained individuals respond to exercise with a faster and more pronounced change in Tsk than untrained individuals. Therefore, it can be assumed that a more pronounced decrease in Tsk during exercise may indicate a more efficient redistribution of blood to the working muscles, which coincides with a slower increase in LA. This could be confirmed by the research by Moreira et al.^26^, in which judokas were assessed during incremental exercise. The authors revealed that the greater the decrease in Tsk in the fifth (r = 0.66, *p* = 0.001) and the 10th (r = 0.55, *p* = 0.001) minutes after exercise, the lower the level of LA after exercise. However, one should consider the specificity of the judo test with alternating 2-min exercise bouts and 1-min rest intervals. Unfortunately, Moreira et al.^26^ did not measure Tsk and LA during exercise, and the first post-exercise measurement was made 5 min after the end of the test. In particular, those authors correlated LA with total body Tsk, with the latter significantly decreasing compared to pre-exercise measurement.

In our research, we observed that from the 10^th^ minute of recovery, higher Tsk levels were correlated with lower LA levels. This could support the observation that a more efficient post-exercise blood redistribution from muscles to skin is associated with faster decreases in LA levels. However, it is not known whether these phenomena are directly related to each other by any physiological or metabolic mechanisms. Of course, we cannot exclude the effect of sweating on the Tsk regulation, which may vary between individuals^40^. Future studies should take into account the role of sweating when interpreting Tsk changes. To sum up, it can be assumed that people with greater or specific exercise capacity show better redistribution of blood between the skin and muscles and/or a more efficient sweating mechanism during exercise and post-exercise recovery. This is manifested by a greater reduction in Tsk during exercise associated with strong blood transfer to the working muscles^11^ with a co-occurring lower LA concentration. In the case of post-exercise recovery, apart from the impact of sweating, a higher Tsk may be associated with strong blood transfer from the skeletal muscles to the skin, which may indicate high blood perfusion in tissues for thermoregulation. The co-occurrence of low LA levels and Tsk in individuals with high exercise capacity suggests that it is possible to estimate training status based on the course of changes in Tsk. Since the methodology we used does not allow us to explain the cause-and-effect relationship of the co-occurrence of parallel changes in Tsk and LA levels, further research is needed to explore this phenomenon.

### Limitations

Among the limitations of the study, we highlight that we have not evaluated the core temperature and the impact of sweating on thermoregulation processes. Furthermore, the conclusions of our research apply only to highly trained athletes.

## Conclusions

During incremental exercise to exhaustion, a significant decrease in Tsk of the lower limbs is observed, followed by rewarming during recovery. The pattern of change in Tsk is related to the training profile. In endurance athletes, Tsk is lower throughout the range of exercise intensity, especially at exhaustion and during post-exercise recovery, while in sprint athletes, Tsk stabilizes between moderate intensity and exhaustion, rising more slowly after exercise. There is a positive correlation between Tsk and blood lactate during exercise at different intensities and the relationship is reversed during recovery. Furthermore, Tsk depends on body composition, i.e., people with higher lean body mass and lower body fat have higher Tsk during both exercise and recovery, suggesting a more efficient heat dissipation. Our study suggests that lower Tsk during exercise, higher Tsk during recovery, higher lean body mass, and lower body fat are associated with higher aerobic capacity in highly trained athletes.

## Data Availability

The datasets generated during and/or analysed during the current study are not publicly available due to use in further analysis, but are available from the corresponding author on reasonable request.
